# Novel Tissue‐Specific Mechanism of Regulation of Angiogenesis and Cancer Growth in Response to Hyperglycemia

**DOI:** 10.1161/JAHA.112.005967

**Published:** 2012-12-19

**Authors:** Sanghamitra Bhattacharyya, Kristina Sul, Irene Krukovets, Carla Nestor, Jianbo Li, Olga Stenina Adognravi

**Affiliations:** 1 Department of Molecular Cardiology and Joseph J. Jacob Center for Thrombosis and Vascular Biology, Lerner Research Institute, Cleveland Clinic, Cleveland, OH (S.B., K.S., I.K., C.N., J.L., O.S.A.)

**Keywords:** complications of diabetes, microRNA, thrombospondin‐1, translational regulation

## Abstract

**Background:**

Hyperglycemia is an independent risk factor for the development of vascular diabetic complications, which are characterized by endothelial dysfunction and tissue‐specific aberrant angiogenesis. Tumor growth is also dependent on angiogenesis. Diabetes affects several cancers in a tissue‐specific way. For example, it positively correlates with the incidence of breast cancer but negatively correlates with the incidence of prostate cancer. The tissue‐specific molecular mechanisms activated by hyperglycemia that control angiogenesis are unknown. Here we describe a novel tissue‐ and cell‐specific molecular pathway that is activated by high glucose and regulates angiogenesis.

**Methods and Results:**

We have identified microRNA 467 (miR‐467) as a translational suppressor of thrombospondin‐1 (TSP‐1), a potent antiangiogenic protein that is implicated in the pathogenesis of several diabetic complications. miR‐467 was upregulated by hyperglycemia in a tissue‐specific manner. It was induced by high glucose in microvascular endothelial cells and in breast cancer cells, where it suppressed the production of TSP‐1 by sequestering mRNA in the nonpolysomal fraction. Mutation of the miR‐467 binding site in TSP‐1 3′ UTR or miR‐467 inhibitor relieved the translational silencing and restored TSP‐1 production. In in vivo angiogenesis models, miR‐467 promoted the growth of blood vessels, and TSP‐1 was the main mediator of this effect. Breast cancer tumors showed increased growth in hyperglycemic mice and expressed higher levels of miR‐467. The antagonist of miR‐467 prevented the hyperglycemia‐induced tumor growth.

**Conclusions:**

Our results demonstrate that miR‐467 is implicated in the control of angiogenesis in response to high glucose, which makes it an attractive tissue‐specific potential target for therapeutic regulation of aberrant angiogenesis and cancer growth in diabetes.

## Introduction

Hyperglycemia is an independent risk factor for the development of diabetic vascular complications characterized by aberrant angiogenesis. Numerous reports not only establish a connection between glucose level and the development of micro‐ and macrovascular complications,^1–4^ but they also implicate glucose in the regulation of many endothelial genes related to abnormal angiogenesis.^5^ However, specific molecular mechanisms of glucose effects remain poorly studied.

Changes in angiogenesis in diabetic patients are tissue‐ and organ specific: aberrant diabetic angiogenesis is characterized by increased neovascularization in one organ (eg, the retina) and decreased angiogenesis in others (eg, skin and myocardium) that can be observed in the same patient at the same time. Cancer is another pathology directly dependent on angiogenesis. It is not surprising that diabetes is positively or negatively associated with several cancers (it positively correlates with hepatic, pancreatic, colon, endometrial, breast, and bladder cancer incidence and prognosis, but negatively with prostate cancer). Thus, both angiogenesis in organs and tumor growth are regulated in a tissue‐specific manner in patients with diabetes. In this report, we describe a novel molecular pathway of regulation of angiogenesis by high glucose: tissue‐specific upregulation of microRNA 467 (miR‐467) and the translational silencing of antiangiogenic protein thrombospondin‐1 (TSP‐1) controlled by miR‐467. This mechanism may explain how hyperglycemia contributes to tissue‐specific aberrant angiogenesis in organs and tumors. The goals of this study were to characterize this novel mechanism at the molecular level, to demonstrate its cell‐ and tissue specificity in vitro and in vivo, and to explore the possibility of regulating hyperglycemia‐induced angiogenesis in vivo by blocking miR‐467 in cancers.

Regulation of angiogenesis is often mediated by controlling the expression of TSP‐1, which is one of the most potent endogenous inhibitors of angiogenesis.^6–8^ TSP‐1 production is regulated by miRNA in tumors.^9–11^ TSP‐1 has been implicated in the development of diabetic complications,^12–13^ which makes it a potential therapeutic candidate for the treatment of diabetes‐induced vascular pathologies. Recently, we described the high‐glucose‐induced, 3′ UTR–mediated translational silencing of TSP‐1 production in microvascular endothelial cells (ECs).^14^ Now we report that miR‐467 is the hyperglycemia‐responsive regulator directly responsible for the translational silencing of TSP‐1 mRNA in a cell‐type‐ and tissue‐specific manner. This molecular pathway is cell‐ and tissue specific and regulates hyperglycemia‐induced angiogenesis and cancer growth.

## Methods

### Cell Culture for In Vitro Experiments

RF/6A, C166, EOMA, EMT6, and RM1 cells were from ATCC. Human umbilical vein ECs (HUVECs) and bovine aortic ECs (BAECs) were kindly provided by Dr DiCorleto (Cleveland Clinic), and human dermal microvascular ECs (HDMECs) and human aortic ECs (HAECs) were from Cambrex.

RF/6A cells were maintained in DMEM/F‐12 medium with 10% fetal bovine serum, and the HUVECs were cultured in MCDB131 (Sigma) supplemented with 15% fetal bovine serum (HiClone), 80 mg/L heparin (Sigma), and endothelial cell growth supplement (ECGS) purified from bovine brain tissue. For transfections, cell passage numbers 2 to 8 were used. Cells were grown in 6‐ or 24‐well clusters (depending on the type of experiment) until they became confluent.

Cells were preincubated in low‐glucose (5 mmol/L) medium (Life Technologies) for 24 hours and transferred into 30 mmol/L cell culture grade d‐glucose, l‐glucose, or sorbitol (Sigma).

### Plasmid Constructs

The complete 5′ and 3′ UTRs of human TSP‐1 were used for plasmid construction. The plasmid constructs for transfection included the full 5′ immediately upstream and the full 3′ UTR of TSP‐1 immediately downstream of the luciferase cDNA. All constructs were cloned into a control pGL3 vector (Promega; described in reference ^14^). Plasmids were purified using a Qiagen HiSpeed Plasmid Purification Kit.

### miRNA Mimics and Inhibitors

miRNA mimics and inhibitors (antagomiRs) were purchased from Thermo Scientific and dissolved in DEPC‐treated water to yield a stock concentration of 20 μmol/L. Cholesterol‐conjugated miR‐467a was modified by tagging of the fluorophore DY547 at the 5′ end of the sense strand and a cholesterol moiety at the 3′ end of the antisense strand with the commercially available mimic of miR‐467a (C‐310732‐03‐005). The control oligonucleotide, the commercially available mimic negative control (CN‐001000‐01‐05), was modified in the same manner.

### Transient Transfections and Luciferase Assay

Cotransfections were carried out using the plasmids in combination with either mimic or inhibitor (100 nmol/L) in 24‐well clusters using Lipofectin (Invitrogen) according to the manufacturer's instruction. The control ribooligonucleotides were used at the same concentration. After 6 hours of transfection, half the cells were transferred into low‐glucose, low‐serum media (DMEM with 5 mmol/L glucose and 2% FBS), and the other half were stimulated with high glucose (same medium supplemented with 30 mmol/L glucose). Cells were harvested and lysates prepared 24 and/or 48 hours later, as indicated for each experiment. Luciferase activity was measured in 100 μg of total protein using reagents from Promega and following the manufacturer's protocol. pGl3 control was used as a control vector to study the effect of TSP‐1 UTRs.

### Western Blot

RIPA buffer (Pierce, Thermo Fisher Scientific, Rockford, IL, USA) supplemented with Protease Inhibitor Cocktail (Roche, Basel, Switzerland) was used to extract total protein from RF/6A cells cotransfected with the UTR constructs and the mimic or inhibitor reagents. Cells stimulated with 30 mmol/L glucose were first washed with 1× ice‐cold PBS and then incubated with RIPA buffer for 15 minutes on ice. Cells were then scraped, collected in ice‐cold centrifuge tubes, and vortexed for 15 seconds. The tubes were then allowed to stand in ice for 30 minutes and centrifuged at 10 000*g* for 20 minutes. Supernatants were collected, and the protein concentration was measured using a Biorad Dc Protein Assay Reagent Kit. Thirty micrograms of total protein was resolved in 10% SDS‐PAGE along with Benchmark Protein Standards (Invitrogen) at 125 V. Resolved proteins were transferred onto a PVDF membrane (Pall Corporation) for 1 hour at 4°C at a constant 100 V. TSP‐1 protein was detected by Western Blot using anti‐TSP‐1 antibody (Labvision) as previously described.^15–17^ The membrane was also probed for β‐actin to assure equal protein loading.

### RNA Extraction

Cells were harvested and lysed using Trizol reagent (Invitrogen) and processed according to the manufacturer's instructions.

### RNA Fractionation

Polysomal and nonpolysomal fractions were prepared on the 30% sucrose cushion as described previously.^14^ Briefly, cells were lysed in polysome lysis buffer, and the fractions were separated by centrifugation on the 30% sucrose cushion. The pellet contained polysomes, the supernatant contained the nonpolysomal fraction.

### Real‐Time RT‐PCR

Total RNA was extracted from cells as described above. Two micrograms of the total RNA was used to synthesize first‐strand cDNA using reagents and the protocol from the Superscript First Strand Synthesis System for RT‐PCR (Invitrogen). The conditions and primers used to measure TSP‐1 and luciferase mRNA levels were described previously.^14^

To measure miRNA levels, 1 μg of total RNA was first polyadenylated followed by first‐strand cDNA synthesis using the protocol for NCode miRNA First‐Strand cDNA Synthesis and a qRT‐PCR kit (Invitrogen). Real‐time PCR amplification was performed with reagents from the same kit. The miRNA sequence‐specific primers used for PCR were purchased from Invitrogen, and the amplification cycles were set according to the instructions outlined in the kit. Ct values were determined as described previously.^14^ Primers for 5s rRNA (Ambion) were used as the housekeeping control RNA.

The products of RT‐PCR synthesized in the process of miR‐467 detection were cloned into pGEMT‐Easy (Promega) and sequenced to confirm that a small RNA with the sequence of mature miR‐467 was detected in these reactions.

### Northern Blotting to Detect miR‐467

RF/6A cells were transfected transiently with miR‐467a to provide a positive control, and both transfected and untransfected cells were then stimulated with 30 mmol/L glucose for 48 hours. MicroRNA was isolated from both transfected and untransfected glucose‐stimulated cells using an miRVana miRNA Isolation Kit (Ambion). The concentration was assessed by a UV absorbance ratio of 260/280 nm. Ten micrograms of the purified RNA was resolved in a 15% denaturing polyacrylamide gel in 1× TBE at a constant current of 40 mA. Decade Markers (Ambion) labeled with γ^32^‐P were diluted (1:50) and resolved adjacent to the samples to correctly determine the size of the small target RNA. The resolved samples were then transferred to a nylon membrane (Gene Screen Plus, Perkin Elmer) by capillary blotting for 16 hours at room temperature in 20× SSC. The transferred membrane was washed with 2× SSC, air‐dried, and UV cross‐linked.

LNA modified ribooligonucleotides (CGCATATACATGCAGGCACTTA, Exiqon, Denmark) complimentary to the target miR‐467a and the Decade Markers were labeled by γ^32^‐P (Perkin Elmer). Unincorporated nucleotides were removed following the protocol provided in instructions for miRVana Probe and Marker Kit (Ambion). Labeling efficiency was determined by the Liquid Scintilation System (Beckman) and represented as counts per minute (cpm) in 1 ml. Probes with a count of 5×10^6^ cpm/mL were used for hybridization. Perfect Hyb Plus Hybridization Buffer (Sigma) was used for miRNA Northern blot analysis. The UV fixed membrane was first prehybridized using 8 mL of this buffer at 65°C for 1 hour, followed by hybridization with the LNA‐modified probe for 16 hours at room temperature. The hybridized membranes were washed twice for 10 minutes with 2× SSC, 0.1% SDS at room temperature before exposing them to Kodak Biomax MR Film. The films were incubated at −80°C to develop signals. Band intensities were quantified using Image J software (http://rsbweb.nih.gov/ij).

### Construction of Point Mutations and Deletion Mutants

Plasmids with the chimeric construct 5′ UTR‐luc‐3′ UTR at a concentration of 20 ng/μL, was taken as the template for creating the point mutant and the deletion mutant. Stratagene's Quick Change Lightning Site Directed Mutagenesis Kit was used according to the manufacturer's instructions. The primers for creating the mutants were purchased from IDT:

#### Point mutations

Forward primer 5′ AGCTGATTAACCCATGTAAATAGG**A**AC**C**TAAATAGAAGCAGG and reverse primer 5′ CCTGCTTCTATTTAGGTTCCTATTTACATGGGTTAATCAGCT were used. An insertion of 1 nucleotide occurred in the sequence of the binding site, and we decided to preserve it as a third unplanned point mutation. The final sequence of the miR‐467a binding site in 3′ UTR was 5′ CATG**G**TAAATAGG**A**AC**C**TAA instead of the wild‐type 5′ CATGTAAATAGGCACTTAA.

#### Deletion mutant

Forward primer 5′ CATGATGCTGACTGGCGTTAGCTGATTAAATAGAAGCAGGAAAG and reverse primer 5′ CTTTCCTGCTTCTATTTAATCAGCTAAAGCCAGTCAGCATCATG were used.

The PCR products were digested by DpnI enzyme and transformed using XL‐10 Gold Ultracompetent cells. One hundred fifty to two hundred microliters of the transformed cells after a 1‐hour recovery with fresh NZY broth was spread on LB‐agar plates supplemented with ampicillin (100 μg/mL), X‐gal (80 μg/mL), and IPTG (20 mmol/L) and incubated overnight at 37°C. A control plate that contained transformed pWhiteScript in place of the plasmid was used to calculate the mutation frequency. Sequencing of colonies was done at the Genomics Core of Cleveland Clinic Foundation using primers (T7, SP6, and internal primers that were previously used to sequence the 3′ UTR). Positive clones lacking the 18‐nt target miR‐467a sequence in 3′ UTR were selected for transfection experiments.

### Stable Expression of miR‐467 Antisense Antagonist in EMT6 Cells

The cells were transfected with either pRNAT‐CMV3.2Neo expressing the antisense oligonucleotide insert (GATCCCCGCATATACATGCAGGCACTTATTTTTTGGAAG) or a negative control oligonucleotide (GATCCCAAATCCTTTAGACCGAGCGTGTGTTTTTTGGAAG). The design of the inserts was based on that described in reference ^18^. Multiple stable clones were selected, and in these clones we tested (1) the expression of the fluorescent tag from the vector and (2) the effect of the antagonist on the level of a reporter protein (luciferase) produced from the fusion construct of luciferase and TSP‐1 UTR that was transiently transfected into these clones. Four clones expressing the antagonist and 4 clones expressing the control oligonucleotide were selected on the basis of the equal expression of the fluorescence tag and equal prevention of the effect of high glucose on luciferase production. These clones were propagated separately, and the cells from 4 clones were mixed in equal amounts before injection into the animals. The injections and tumor analyses were performed as described below.

### Experimental Animals

Seven‐ to eight‐week‐old C57/BL6J male mice (Jackson Laboratories) were used in the described experiments (n≥6 per experimental point or condition). They were housed and cared for in the AAALAC‐approved animal facilities of Cleveland Clinic. All animal studies were approved by the Institutional Animal Care and Use Committee, and all the experiments were conducted strictly in accordance with National Institutes of Health and institutional guidelines. A ketamine/xylazine cocktail (80 mg/kg IP) was used for anesthesia to immobilize the mice for subcutaneous Matrigel or cancer‐cell injections and blood collection from the tail vein.

*Lepr*^*db/db*^ and control *Lepr*^*db/+*^ male mice were purchased from Jackson Laboratories and used at 12 weeks of age.

### Induction of Diabetes in Mice

The male wild‐type C57BL/6 mice were given intraperitoneal streptozotocin (STZ; in 20 mmol/L citrate buffer, pH 4.6) injection following the procedure proposed by Jackson Laboratories (50 mg/kg for 5 consecutive days). Age‐matched controls received only citrate buffer injections. Blood glucose was measured from samples taken from the tail vein starting 48 hours after the STZ injection using the AlphaTrak Blood Glucose monitoring system. Mice with blood glucose >250 mg/dL were selected for experiments.

In *Lepr*^*db/db*^ mice and the control lean nondiabetic *Lepr*^*db/+*^ mice, blood glucose levels were measured on the day they were euthanized at the end of the experiment and ranged from 141 to 333 mg/dL in *Lepr*^*db/db*^ mice and from 91 to 131 mg/dL in control *Lepr*^*db/+*^ mice.

### In Vivo Angiogenesis Assay

Mice were anesthetized, and the abdominal areas were shaved and swabbed with 70% ethanol. Mice were injected subcutaneously into the right flank with 500 μL of Matrigel Matrix Basement Membrane (BD Biosciences) supplemented with bFGF (750 ng/mL, R&D Systems), heparin (26 U/mL, Sigma), and miR‐467 mimic or control ribooligonucleotide with a 5′ fluorescence tag and a 3′ cholesterol tag (Thermo Scientific). Purified TSP‐1 was purchased from R&D and added to Matrigel at a final concentration of 10 μg/mL. Subcutaneous plugs were removed 7 days later.

### Mouse Tumor

EMT6 or RM1 cells 1.5×10^6^ were injected subcutaneously to the control or hyperglycemic mice 1 week after the mice became hyperglycemic. The tumors were excised 7 days (RM1) or 9 days (EMT6) later from euthanized mice and weighed, frozen in liquid nitrogen until processed, or processed immediately to isolate RNA.

### Staining of the Slides and Image Analyses

Frozen sections were stained with H&E or specific antibodies using Vecta Stain ABC Kit according to the manufacturer's instructions. Biotinylated rat anti‐mouse CD31 (1:100; Abcam) and rabbit polyclonal anti‐α‐actin (1:150; Abcam) were used to visualize EC and smooth muscle cells/pericytes, respectively. Mouse monoclonal anti‐TSP‐1 Ab 4 (clone 6.1) from Thermo Scientific (1:50) was used to assess TSP‐1 protein levels. Anti‐CD3 and anti‐CD8 antibodies were from Abcam and Novus Biological, respectively.

To Analyze Matrigel plugs and prostate cancer tumors, we used a fluorescence microscope at ×10 to ×25 magnification by a blinded investigator. Fifteen to 20 random fields from at least 2 sections of each plug (from ≥9 animals) were quantified. Quantification was carried out using Image Pro 6.1.3.

To analyze breast cancer tumors, images of staining were acquired using a Leica DMR 4000B upright microscope (Heidelberg, Germany) fitted with a single‐slide x‐, y, and z‐motorized stage, a ×20 (dry) objective, a FITC fluorescence filter cube, and a Retiga 2000R CCD digital camera (QImaging, Burnaby, British Columbia, Canada). High‐magnification image fields were raster‐scanned across each cross‐section (Oasis 4i controller, Objective Imaging, Kansasville, MI) and stitched together to form a single high‐resolution, large field‐of‐view (FOV) image (≈289 tiles/mosaic). Each image field was background‐corrected prior to stitching to ensure continuity and minimize chromatic variability. For quantitative analysis, large FOV images were batch‐processed using customized macros and algorithms generated for Image‐Pro Plus 6.1 (Media Cybernetics, Silver Spring, MD). The total area of tumor and the stained area of each cross‐section were then exported to Excel.

### Tumor Cell Proliferation Assay

EMT6 cells stable‐transfected with a vector expressing the antisense antagonist of miR‐467 or a control oligonucleotide (4 clones for each) were plated in 24‐well cell culture clusters (5000 cells/well). Cells were harvested for count or lysed in clusters for DNA amount measurement using CyQuant Proliferation kit (Invitrogen) 24 and 48 hours later.

### Statistical Analysis

All values are expressed as mean±standard error of the mean (SEM). Prior to an analysis, the Shapiro–Wilk normality test was used on each data set, on the original scale, or after transformation, and parametric methods were used when the assumption of normality was met, whereas nonparametric methods were applied if the assumption was violated. One or 2‐way ANOVA was used for data with 1 or 2 factors, respectively, and the Student *t* test was used to compare 2 group means. The nonparametric counterparts were the Friedman rank sum test with stratifications for 2‐way ANOVA, the Kruskal–Wallis test for 1‐way ANOVA, and the Wilcoxon rank sum test for the *t* test. For subgroup multiple comparisons, Tukey's test was applied. For correlation analysis, Spearman's rank correlation test was used because of its relaxed assumptions of normality and linearity. The results were considered statistically significant at α=0.05. All data analyses were done using the statistical software package R (R Development Core Team, http://www.R-project.org).

## Results

### miR‐467 Level Is Upregulated by High Glucose in Microvascular ECs in a Cell‐Type‐Specific Manner

**Figure 1.**
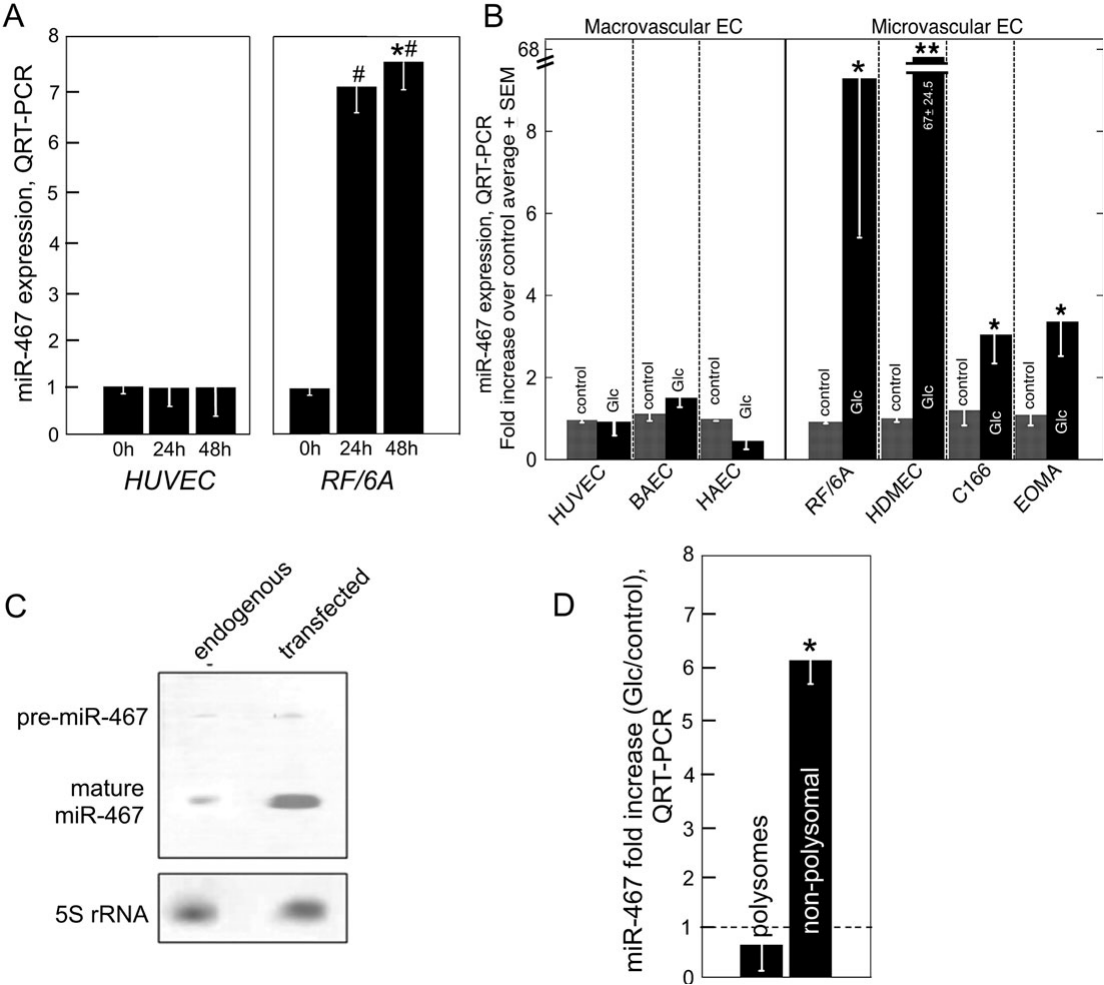
Cell‐type‐specific upregulation of miR‐467 and its association with nonpolysomal RNA in response to high glucose. A, Expression of miR‐467 in HUVECs and RF/6A 24 and 48 hours on high‐glucose stimulation. Real‐time RT‐PCR, mean±SEM, *P<0.05 compared with 0‐hour sample (=1), #*P*<0.05 compared with a similar sample in HUVECs, n=5. B, Cell‐type‐specific expression of miR‐467. BAECs, bovine aortic endothelial cells; HAECs, human aortic endothelial cells; HDMECs, human dermal microvascular endothelial cells; C166 and EOMA, 2 immortalized microvascular EC lines. Twenty‐four‐hour stimulation with 30 mmol/L glucose, real‐time RT‐PCR, mean±SEM, *P<0.05 compared with control (low glucose) sample, HAECs: n=4; HUVECs, RF/6A: n=5; BAECs: n=9; HDMECs: n=11; C166, EOMA: n=13. Levels of miRNA were normalized to 5S levels in each sample. C, Northern blotting: left lane, endogenous miR‐467 was detected in RF/6A stimulated with 30 mmol/L glucose using LNA‐modified probe; right lane, miR‐467 was transfected into RF/6A cells as a positive control. D, Translocation of miR‐467 to the nonpolysomal RNA fraction in microvascular EC RF/6A in response to glucose. Real‐time RT‐PCR, mean±SEM. *P<0.05 compared with miR‐467 levels in the same RNA fraction in low glucose (=1), n=6. Poly indicates polysomal fraction; nonpoly, nonpolysomal fractions; HUVECs, human umbilical vein endothelial cells; ECs, endothelial cells; SEM, standard error of mean; RT‐PCR, reverse‐transcription polymerase chain reaction; BAECs, bovine aortic endothelial cells; HDMECs, human dermal microvascular endothelial cells.

We previously reported that in microvascular endothelial cells (ECs) the inhibition of TSP‐1 protein production occurred despite the upregulation of mRNA, a result of translational silencing in microvascular ECs.^14^ Using the miRBase database (http://www.mirbase.org/), 7 putative binding sites for miRNA were predicted in the TSP1 3′ UTR: miR‐19a, miR‐21, miR‐194, miR‐467, let‐7b, let‐7i, and miR‐101. The expression of these 7 miRNAs was measured in microvascular EC RF/6A and in macrovascular EC HUVEC 24 hours after stimulation with 30 mmol/L glucose (Figure S1). miR‐467 was the only miRNA that had the cell‐type‐specific induction profile in response to glucose that matched the profile of inhibition of TSP‐1 production^14^: miR‐467 was induced after glucose stimulation (*P*<0.05) in microvascular EC RF/6A, but not in HUVEC (Figures 1A and S1). Such an expression profile was unique and specific for miR‐467 in contrast to the other 6 miRNAs, which were not significantly induced by glucose in RF/6A (Figure S1).

In addition to HUVEC and RF/6A, miR‐467 expression was also measured in other types of macrovascular ECs (bovine aortic endothelial cells, BAECs; human aortic endothelial cells, HAECs) and microvascular ECs (human dermal microvascular ECs, HDMECs; and microvascular EC cell lines C166 and EOMA) (Figure 1B). Similar to the cell‐specific pattern of translational silencing of TSP‐1,^14^ miR‐467 was upregulated in microvascular ECs only.

miR‐467 was induced by physiologically active and intracellularly metabolized d‐glucose, physiologically inactive l‐glucose, or sorbitol (Figure S2A). A similar effect was observed previously with TSP‐1 silencing that was a result of increased osmolarity and did not depend on intracellular glucose metabolism.^14^ The induction ranged from 3.5‐fold (d‐glucose) to 6.5‐fold (l‐glucose). The induction by nonmetabolized sugars was significantly higher and time dependent, presumably, because they better maintained higher osmolarity, whereas d‐glucose was consumed over time.

The response to increased d‐glucose or biologically inactive l‐glucose could be observed at concentrations as low as 10 mmol/L (corresponding to 173 mg/dL of blood glucose level) (Figure S2B).

### Detection of Endogenous miR‐467 in Northern Blotting

miR‐467 was detected in glucose‐stimulated RF/6A by Northern blotting (Figure 1C). We used LNA‐modified antisense ribooligonucleotide as a probe. A band of ≈20 bp was detected in microvascular EC RF/6A stimulated with 30 mmol/L glucose. To assure that the band was of the expected size, we transfected the oligonucleotide with the sequence of miR‐467 into RF/6A cells and detected it along with the endogenous miR‐467. As seen in Figure 1C, the transfected miR‐467 oligonucleotide and the endogenous miR‐467 had identical electromobility in the gel. miR‐467 appears to be expressed at a very low level or is undetectable in control cells and tissues; in this experiment, we were unable to detect a visible band in control cells incubated in 5 mmol/L glucose.

### Human miR‐467

Product of RT‐PCR detected in our quantitative analyses described above was cloned into a pGEMT‐Easy vector (Promega) and sequenced. Sequencing has confirmed that a short 22‐nt RNA was detected in these analyses.

In addition to our analyses, Dr Karnik (Cleveland Clinic) has detected miR‐467 and significant changes in its expression in response to specific stimuli in human vascular smooth muscle cells using the expression array approach (personal communication), independently confirming the expression of miR‐467 in human cells.

According to the public databases, a predicted binding site for miR‐467 exists in both mouse and human 3′ UTR of TSP‐1.

### High Glucose Induces Translocation of miR‐467 to the Nonpolysomal Fraction of RNA

On stimulation with high glucose, TSP‐1 mRNA translocates to the nonpolysomal RNA fraction (the fraction containing messages that are not actively translated, as opposed to actively translated messages associated with polysomes and precipitate in the polysomal fractions).^14^ Although circumstantial, translocation of miR‐467 to the nonpolysomal fraction would support a role for this miRNA in translational inhibition. Similar to TSP‐1 mRNA,^14^ miR‐467 was translocated to the nonpolysomal fraction of RNA in microvascular EC RF/6A on stimulation with glucose (Figure 1D). The amount of miR‐467 in the nonpolysomal fraction containing messages that were not actively translated increased by ≈6‐fold, whereas the amount in the polysomal fraction containing actively translated messages did not increase. Consistent with the profile observed for TSP‐1 mRNA in macrovascular ECs stimulated with high glucose (it was found in the polysomal fraction^14^), there was no translocation of miR‐467 to the nonpolysomal fraction in macrovascular ECs (not shown). We confirmed the specificity of the translocation of miR‐467 by analyzing the distribution profile for 2 other miRNAs (Figure S3A): the translocation of miR‐467 was a specific event, not the general trend. To control the quality of RNA samples, we visualized ribosomal RNA in fractions in agarose gel electrophoresis (Figure S3B).

### miR‐467 Controls TSP‐1 Protein Production in Microvascular ECs Stimulated With High Glucose

**Figure 2.**
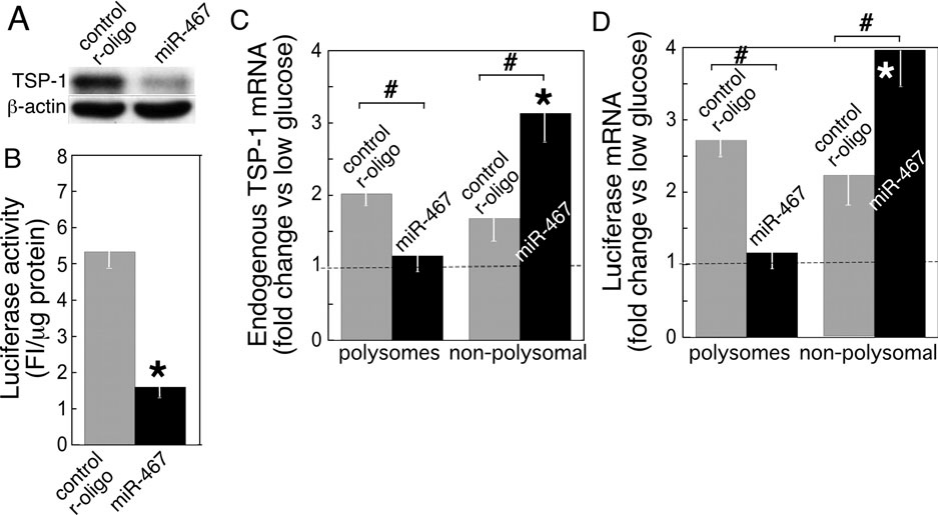
miR‐467 mediates inhibition of TSP‐1 protein production by uncoupling mRNA from polysomes. A, Transfection of RF/6A with miR‐467 mimic results in inhibition of TSP‐1 production. Western blot, anti‐TSP‐1, and anti‐β‐actin antibodies. B, Inhibition of production of the reporter gene in cells cotransfected with miR‐467 mimic and the reporter construct expressing luciferase cDNA fused to TSP‐1 UTR cDNA. Luciferase activity in cell lysates, FI, fluorescence intensity; mean±SEM, n=4, *P<0.05. C and D, miR‐467 mimic induces uncoupling of TSP‐1 mRNA (C) and the chimeric luciferase‐TSP‐1 UTR mRNA (D) from polysomes and their association with the nonpolysomal fraction. mRNA of TSP‐1 and luciferase were detected by real‐time RT‐PCR in each RNA fraction. Mean±SEM, n=4, *P<0.05 compared with low‐glucose sample (=1), #*P*<0.05 compared with cells transfected with a control ribooligonucleotide (control r‐oligo). TSP indicates thrombospondin; UTR, untranslated region; SEM, standard error of mean; RT‐PCR, reverse‐transcription polymerase chain reaction.

To directly confirm the effect of miR‐467 on TSP‐1 protein production, we used a cholesterol‐conjugated miR‐467 mimic^19–20^ as described in Methods. As a negative control, we used a cholesterol‐conjugated ribooligonucleotide with an unrelated sequence that does not have any matches in the UTRs of known genes (Thermo Scientific). The transfection efficiency was >50% in both RF/6A and HUVEC as determined by the live observation of fluorescence from the second fluorescent label (DY547**)** in the transfected cells (Figure S4). Transfection with miR‐467 mimic resulted in a significantly decreased level of endogenous TSP‐1 and the reporter protein expressed from a plasmid containing TSP‐1 UTR compared with the effect of the control ribooligonucleotide (Figure 2A and 2B). In HUVECs, transfection with miR‐467 did not affect protein production (Figure S5A).

### miR‐467 Controls Association of TSP‐1 mRNA With Polysomes

To determine whether miR‐467 is responsible for sequestering TSP‐1 mRNA to the nonpolysomal fraction with untranslated mRNA,^14^ we performed RNA fractionation using lysates from RF/6A transfected either with the miR‐467 mimic or the control ribooligonucleotide (Figure 2C). As shown in Figure 2C, the amount of TSP‐1 mRNA in the nonpolysomal fraction increased in cells transfected with the miR‐467 mimic. The transfection of cells with the control oligonucleotide did not affect TSP‐1 mRNA localization.

To find out whether the effect of miR‐467 on association of mRNA with polysomes depends on the UTR of TSP‐1, we cotransfected RF/6A with the miR‐467 mimic along with a reporter (luciferase) cDNA fused to the UTRs of TSP‐1. Similar to the endogenous TSP‐1 mRNA, the fusion transcript accumulated in the nonpolysomal fraction when cells were cotransfected with the miR‐467 mimic (Figure 2D).

Transfection of HUVECs with miR‐467 mimic did not affect the distribution of TSP‐1 or reporter mRNA between polysomal and nonpolysomal fractions compared with transfection with the control oligonucleotide (Figure S5B and S5C).

### miR‐467 Antagonist Relieves Glucose Inhibition of TSP‐1 Protein Production

**Figure 3.**
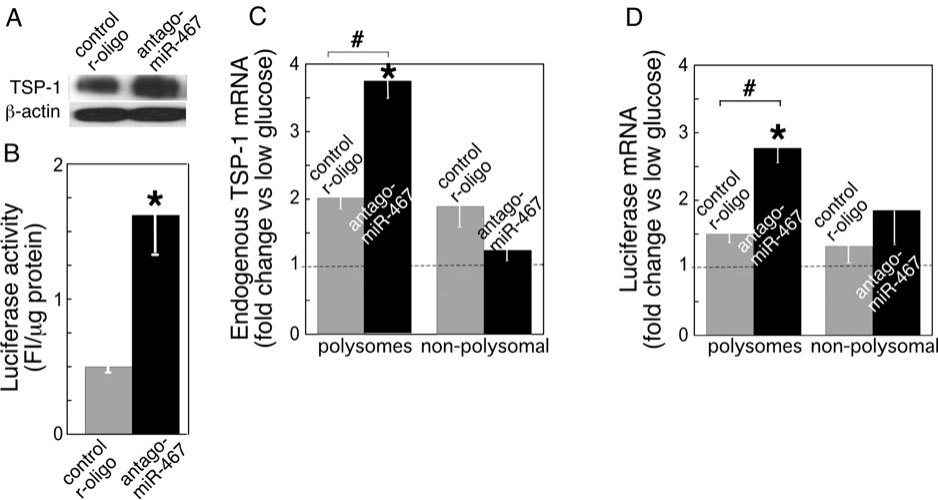
Antagonist of miR‐467 prevents inhibition of TSP‐1 production and mRNA uncoupling from polysomes in response to high glucose. A and B, Transfection of RF/6A with antagomiR‐467 prevents inhibition of production of endogenous TSP‐1 and the luciferase produced from a reporter construct expressing luciferase cDNA fused to TSP‐1 UTR cDNA in response to 30 mmol/L glucose. A, Western blot, anti‐TSP‐1 and anti‐β‐actin antibodies. B, Luciferase activity in cell lysates, FI, fluorescence intensity; mean±SEM, n=4, *P<0.05. C, AntagomiR‐467 prevents sequestering of endogenous TSP‐1 message with the nonpolysomal RNA. Endogenous TSP‐1 mRNA was detected by real‐time RT‐PCR in each RNA fraction. Mean±SEM, n=4, *P<0.05 compared with low‐glucose sample (=1), #*P*<0.05 compared with cells transfected with a control ribooligonucleotide (control r‐oligo). D, AntagomiR‐467 prevents uncoupling of the chimeric luciferase‐TSP‐1 UTR mRNA from polysomes. Luciferase mRNA was detected by real‐time RT‐PCR in each RNA fraction. Mean±SEM, n=4, *P<0.05 compared with low‐glucose sample (=1), #*P*<0.05 compared with cells transfected with a control ribooligonucleotide (control r‐oligo). TSP indicates thrombospondin; UTR, untranslated region; SEM, standard error of mean; RT‐PCR, reverse‐transcription polymerase chain reaction.

We adopted an alternative approach to further confirm that miR‐467 is responsible for the inhibition of TSP‐1 production and the UTR‐mediated translocation of mRNA to the nonpolysomal fraction. We used an antisense inhibitor of miR‐467 (antagomiR, Thermo Scientific) to reverse the effects of glucose in RF/6A. An unrelated ribooligonucleotide (Thermo Scientific) that does not match any known miRNA sequence was used as a negative control. The transfected RF/6A cells were stimulated with high glucose for 48 hours, and TSP‐1 was detected by Western blotting (Figure 3A). In glucose‐stimulated cells transfected with antagomiR‐467, TSP‐1 protein levels were significantly increased compared with the cells transfected with the control oligonucleotide, suggesting that neutralization of miR‐467 by antagomiR relieves the inhibition of TSP‐1 production.

### miR‐467 Antagonist Relieves Inhibition of Reporter Protein Production in Response to Glucose

When antagomiR‐467 was cotransfected with the reporter‐TSP‐1 3′ UTR construct, it increased production of the reporter protein compared with the cells cotransfected with the control oligonucleotide (Figure 3B). We observed a significant recovery of luciferase production in RF/6A cells transfected with antagomiR‐467.

### AntagomiR‐467 Prevents Glucose‐Induced Translocation of Endogenous TSP‐1 mRNA and Luciferase‐TSP1 UTR mRNA to the Nonpolysomal Fraction

The translocation of both endogenous TSP‐1 mRNA and luciferase‐TSP‐1 UTR mRNA to the nonpolysomal RNA fraction in response to glucose stimulation was inhibited when the cells were transfected with antagomiR‐467 (Figure 3C and 3D). AntagomiR‐467 promoted its association with the polysomal fraction of RNA containing actively translated messages.

### Point Mutation or Deletion of miR‐467 Binding Site Relieves Inhibition of Protein Production

**Figure 4.**
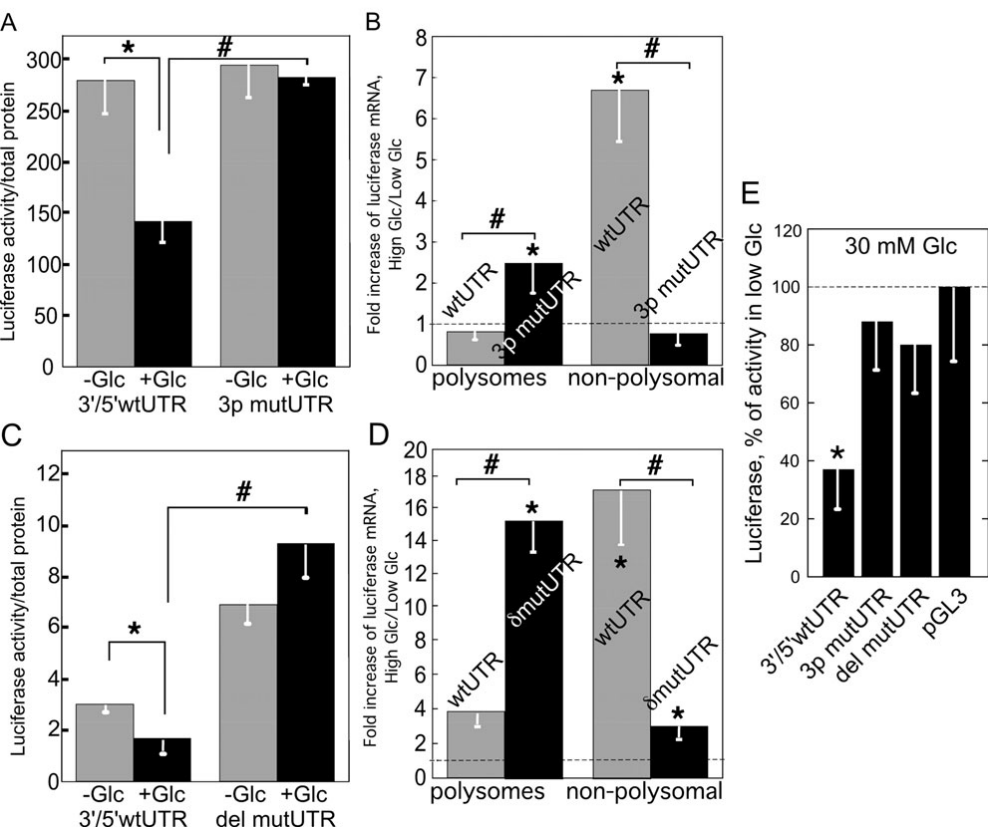
Point mutations or deletion of the target binding site for miR‐467 in 3′ UTR of TSP‐1 prevent inhibition of protein production by high glucose and the uncoupling of mRNA from polysomes. A and C, Luciferase activity, expressed as fluorescence intensity per microgram (FI/μg) of protein, is not downregulated by high glucose in the absence of the miR‐467 target site on the 3′ UTR (A: 3p mut=construct with 3‐point mutations in the binding site; C: dM=deletion mutant construct), mean±SEM, n=6. Construct expressing luciferase cDNA fused to wild‐type (5′‐luc‐3′) TSP‐1 UTR is used as a control. *P<0.05 compared with low‐glucose sample, #*P*<0.05 in comparisons of wild‐type and mutant UTR constructs. B and D, Transcripts of both point mutant and deletion mutant constructs remain associated with the polysomes, mean±SEM, n=6. Luciferase mRNA was detected by real‐time RT‐PCR for wild‐type and mutant transfected cells. *P<0.05 compared with low‐glucose sample (=1), #*P*<0.05 in comparisons of wild‐type and mutant UTR constructs. E, miR‐476 was cotransfected with wild‐type, 3‐point mutant, deletion mutant construct, and control pGL3 vector. Luciferase activity of cells transfected with control oligonucleotide was used as 100% for each of 4 constructs. *P<0.05 compared with low‐glucose sample (100%). TSP indicates thrombospondin; UTR, untranslated region; SEM, standard error of mean; RT‐PCR, reverse‐transcription polymerase chain reaction.

To confirm that the direct binding of miR‐467 to the 3′ UTR of TSP‐1 causes inhibition of protein production, we created 2 mutant constructs. The 3‐point mutant construct had 2 nucleotide bases mutated and 1 nucleotide insertion (see Methods). In the deletion construct, the whole predicted binding site for miR‐467 (18 nucleotides) was deleted from the original construct containing luciferase cDNA fused to the UTR of TSP‐1. These constructs were transfected into microvascular EC RF/6A, and the activity of luciferase was measured in cell lysates 48 hours after stimulation with glucose (Figure 4A through 4D). Luciferase activity was compared with the activity in lysates of cells transfected with the original wild‐type luciferase‐TSP‐1 UTR construct (Figure 4A and 4C). The production of luciferase was significantly inhibited by glucose in cells transfected with the original construct containing the miR‐467 binding site in the TSP‐1 3′ UTR (Figure 4A and 4C). In contrast, there was no inhibition in response to glucose in cells transfected with the mutant constructs.

### Point Mutations or Deletion of the miR‐467 Binding Site From the 3′ UTR of TSP‐1 Prevent Dissociation of mRNA From Polysomes and mRNA Translocation to the Nonpolysomal Fraction

To demonstrate that the binding of miR‐467 to its binding site in the 3′ UTR of TSP‐1 results in uncoupling of TSP‐1 mRNA from polysomes, we fractionated lysates of glucose‐stimulated cells transfected with the point or deletion mutants (Figure 4B and 4D). In glucose‐stimulated cells transfected with the wild‐type construct, the reporter mRNA accumulated mostly in the nonpolysomal fraction. However, in the cells transfected with either of the mutant constructs, most of the luciferase‐TSP‐1 UTR transcript was present in the polysomal fraction associated with the actively translated messages (Figure 4B and 4D).

When the cholesterol‐modified miR‐467 mimic was cotransfected with either of the 2 mutant constructs (Figure 4E), miR‐467 failed to suppress production of the reporter protein from the mutant constructs, confirming that the direct binding of miR‐467 to 3′ UTR is required for translational silencing. The activity of luciferase produced from the wild‐type construct in cells cotransfected with miR‐467 was <40% of the activity in cells cotransfected with a control oligonucleotide. However, the activity of luciferase produced from either of the mutant constructs or from the original pGL vector lacking the UTR of TSP‐1 was similar in the cells cotransfected with miR‐467 and the cells cotransfected with the control oligonucleotide.

### Effect of miR‐467 on Angiogenesis

**Figure 5.**
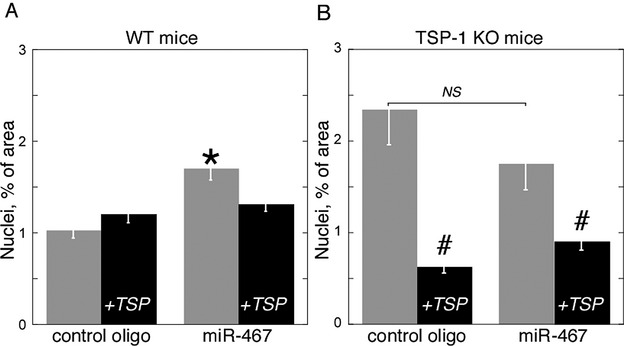
miR‐467 increases angiogenesis in vivo by silencing TSP‐1. Matrigel without exogenous TSP‐1 and Matrigel containing 10 μg/mL purified TSP‐1, both containing either miR‐467 or control oligonucleotide was injected to wild‐type (A) or TSP‐1‐knockout (B) mice; TSP‐1=Matrigel with 10 μg/mL TSP‐1. Sample size: wild type (left panel), 108 fields from 6 mice (control oligo) and 115 fields from 6 mice (miR‐467); wild type+TSP‐1, 115 fields from 6 mice (control oligo) and 113 fields from 6 mice (miR‐467); knockout (right panel), 102 fields from 5 mice (control oligo) and 78 fields from 4 mice (miR‐467); knockout+TSP‐1, 99 fields from 5 mice (control oligo) and 76 fields from 4 mice (miR‐467). Mean±SEM, *P<0.05 compared with the plug containing control oligo, #*P*<0.05 compared with the plug without exogenous TSP‐1 or containing TSP‐1 (+TSP). NS indicates not significant; WT, wild type; TSP, thrombospondin; UTR, untranslated region; SEM, standard error of mean.

To demonstrate the effect of miR‐467 on in vivo angiogenesis, we tested the effect of the cholesterol‐conjugated miR‐467 mimic embedded in Matrigel in a mouse model. Matrigel mixed with bFGF (750 ng/mL) and either cholesterol‐conjugated miR‐467 mimic or cholesterol‐conjugated control ribooligonucleotide was injected subcutaneously into wild‐type C57BL/6 mice. The Matrigel plugs were excised from euthanized animals 7 days later, fixed, and processed as described in Methods. The sections were stained with hematoxylin to determine the total number of cells per section. Imaging and quantification were performed by a blinded investigator as described in Methods. The miR‐467 mimic increased the number of cells in the plug, suggesting increased proangiogenic activity in these plugs (Figure 5A).

### TSP‐1 Is the Main Target of miR‐467 and the Main Mediator in Regulation of Angiogenesis

Exogenous purified TSP‐1 was added to the Matrigel solution at a concentration of 10 μg/mL immediately before the injection (Figure 5A and 5B). TSP‐1 in Matrigel decreased angiogenesis caused by miR‐467 compared with the plugs without exogenous TSP.

When TSP‐1‐knockout (KO) mice (Jackson Laboratories) were used in the same experimental design (Figure 5B), miR‐467 failed to increase angiogenesis in the plug, and the exogenous TSP‐1 had a profoundly significant antiangiogenic effect in all animals.

### Expression of miR‐467 in Tissues From Hyperglycemic Mice

To demonstrate the tissue‐specific expression of miR‐467 in response to hyperglycemia, levels of miR‐467 were measured by quantitative RT‐PCR in tissues from STZ‐treated hyperglycemic mice (fasting glucose >250 mg/dL): in hearts, in lungs, in kidneys, and in Matrigel plugs containing only vascular cell types (Figure S6A). miR‐467 was upregulated by hyperglycemia in a tissue‐specific manner in microvessels of the Matrigel plugs, lung, and kidney, but not in the heart tissue.

### Expression of miR‐467 in Glucose‐Stimulated Cancer Cells

To find out whether the tissue‐specific effect of high glucose on miR‐467 expression can be observed in breast cancer cells and prostate cancer cells, we measured miR‐467 levels in EMT6 (breast cancer) and RM1 (prostate cancer) cultured cells 3, 6, and 24 hours after a single exposure to 30 mmol/L glucose. As shown in Figure S6B, there was no effect of high glucose on miR‐467 in RM1 cells, but EMT6 cells responded by up to a 5‐fold upregulation of miR‐467 levels 3 and 6 hours after stimulation. miR‐467 level decreased to basal after 24 hours, presumably because of the decreased level of glucose in the media of these metabolically active cells (glucose levels in the media were <70 mg/kg [<4 mmol/L] at 24 hours).

### Prostate Cancer Growth in STZ‐Treated Hyperglycemic Mice

RM1 (mouse prostate) cancer cells were injected subcutaneously into STZ‐treated C57Bl/6 mice with blood glucose levels >250 mg/dL. Tumors were excised a week later, weighed, and processed for RNA and protein isolation or for immunohistochemistry to measure TSP‐1 protein and mRNA expression, miR‐467 level, and CD31 level.

**Figure 6.**
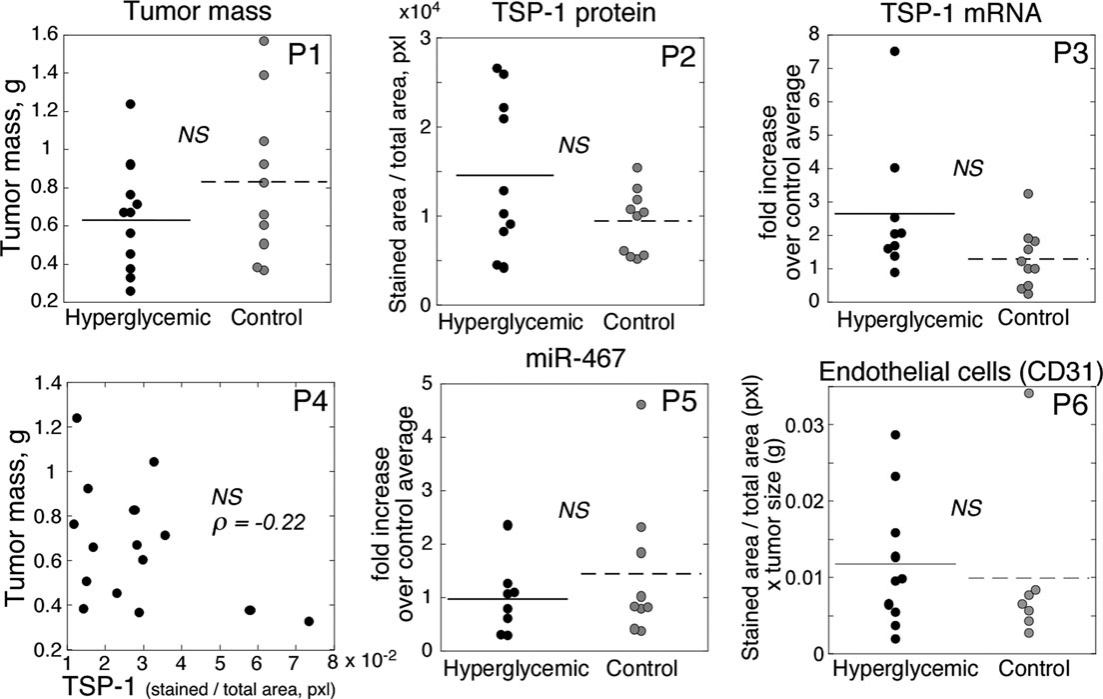
Prostate cancer growth in hyperglycemic mice. RM1 cancer cells were injected subcutaneously to hyper‐ or normoglycemic mice as described in Methods. Tumors excised a week later were weighed (P1, n=10 in control group, n=11 in hyperglycemic group), stained with anti‐TSP‐1 and anti‐CD31 antibodies, and TSP‐1 mRNA and miR‐467 levels were measured by QRT‐PCR. Levels of proteins and RNA for each individual mouse are shown on the graph. In P2, P3, P5, and P6, n=6 to 10 because of an insufficient amount of tissue to accomplish all the analyses in some samples. For the analyses of immunohistochemical staining (P2 and P6), 5 to 11 fields from each plug were quantified, depending on the size of the tumor (total number of fields >85 for each protein in either the diabetic or the normoglycemic mice). In P4, data from both the normoglycemic and hyperglycemic groups are combined, and n=15. NS indicates difference between means is not statistically significant; TSP, thrombospondin; RT‐PCR, reverse‐transcription polymerase chain reaction.

The size of prostate tumors tended to be smaller in hyperglycemic mice (Figure 6, P1), but the difference did not reach statistical significance. Both TSP‐1 mRNA expression and TSP‐1 protein levels (Figure 6, P2 and P3) tended to be increased in hyperglycemic animals. A trend of correlation between TSP‐1 level and individual tumors size (Figure 6, P4) was suggested by the data but was not significant. Consistent with the lack of downregulation of TSP‐1 protein level and similar to the in vitro response to high glucose (Figure S6B), there was no increase in miR‐467 level (Figure 6, P5). In this cancer model, there were no correlations between tumor mass and TSP‐1 level, miR‐467 and TSP‐1 levels, or miR‐467 and tumor mass levels in individual animals (data not shown).

### Breast Cancer in STZ‐Treated Hyperglycemic Mice

**Figure 7.**
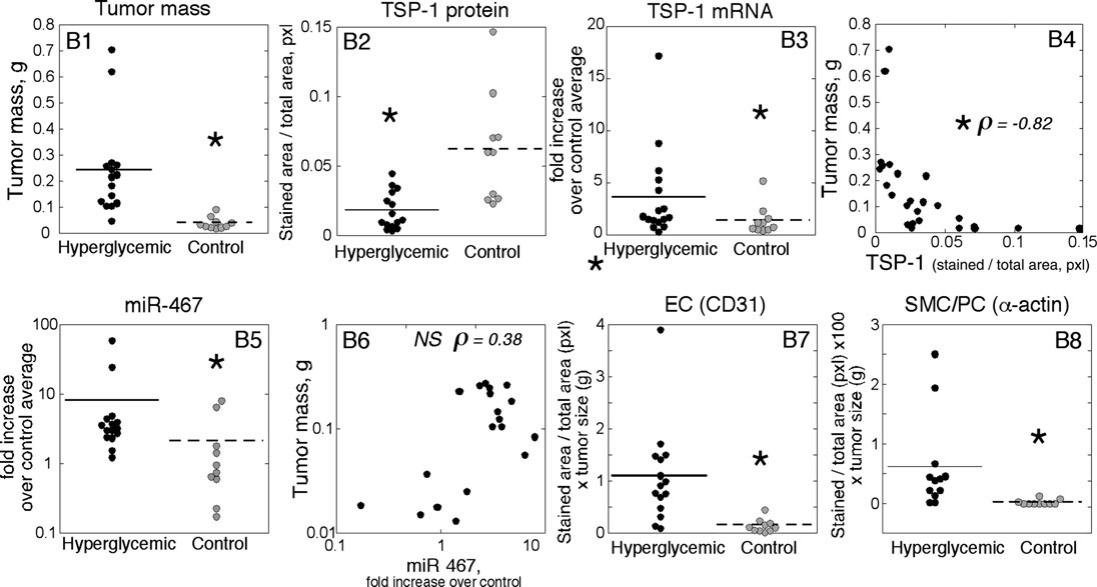
miR‐467 regulates breast cancer growth in STZ‐treated hyperglycemic mice. EMT6 cancer cells were injected subcutaneously into STZ‐treated hyperglycemic or normoglycemic mice as described in Methods. Tumors excised a week later were weighed (B1), stained with anti‐TSP‐1 (B2), anti‐CD31 (B7), and α‐actin antibodies (B8). TSP‐1 mRNA (B3) and miR‐467 (B5) levels were measured by QRT‐PCR. Levels of proteins and RNA for each individual mouse are shown on the graph. A, STZ‐induced hyperglycemia, n=10 in control group and n=16 in hyperglycemic group. In B4, B6, and B7, data from both the normoglycemic and hyperglycemic groups were combined, n=25. For the analyses of immunohistological staining (B2 and B8), large field‐of‐view (FOV) images were generated (see Methods for details) from each tumor sections. *P<0.05 in comparisons between normoglycemic (control) and hyperglycemic mice. In B4 and B6, *P of correlation <0.05; NS indicates no statistically significant correlation; STZ, streptozotocin; TSP, thrombospondin; RT‐PCR, reverse‐transcription polymerase chain reaction.

EMT6 cells 1.5×10^6^ were injected subcutaneously into either normoglycemic or hyperglycemic wild‐type C57Bl/6 mice treated with STZ (Figure 7). The tumors were excised and weighed a week later (Figure 7, B1). TSP‐1 mRNA and protein, miR‐467, CD31, and α‐actin levels were measured and quantified to establish correlations between hyperglycemia, miR‐467 and TSP‐1 protein levels, tumor size, and proangiogenic activity. Unlike prostate cancer tumors (Figure 6, P1), breast cancer tumors (Figure 7, B1) were significantly larger in hyperglycemic mice. TSP‐1 mRNA expression was significantly increased in breast cancer tumors of hyperglycemic animals. Unlike in prostate cancers, in which the increased TSP‐1 mRNA resulted in increased protein levels (Figure 6, P2 and P3), in breast cancers TSP‐1 protein levels were dramatically reduced despite high mRNA expression (Figure 7, B2 and B3). There was a negative correlation between TSP‐1 level and individual tumors size (Figure 7, B4). A significant increase in miR‐467 level was found in breast cancer tumors (Figure 7, B5). miR‐467 level tended to correlate with tumor mass in individual mice (Figure 7, B6). CD31 level (Figure 7, B7) and smooth muscle actin level (Figure 7, B8) were significantly higher in hyperglycemic tumors (Figure 7, B7), indicating increased angiogenesis and consistent with the increased tumor mass.

Tumor mass positively correlated with blood glucose levels in individual mice in the breast cancer model in STZ‐treated mice, and miR‐467 level tended to correlate with blood glucose level (Figure S7). As was expected, TSP‐1 level negatively correlated with blood glucose level (Figure S7).

To exclude the effect of different states of the immune system in hyperglycemic and normoglycemic mice, the number of CD3‐ and CD8‐positive cells in tumors was evaluated (Figure S8). The number of CD8‐positive cells was equal, and the number of CD3‐positive cells was not significantly different.

### Expression of miR‐467 Inhibitor in Breast Cancers Prevents Stimulation of Their Growth by Hyperglycemia in STZ‐Treated Mice

**Figure 8.**
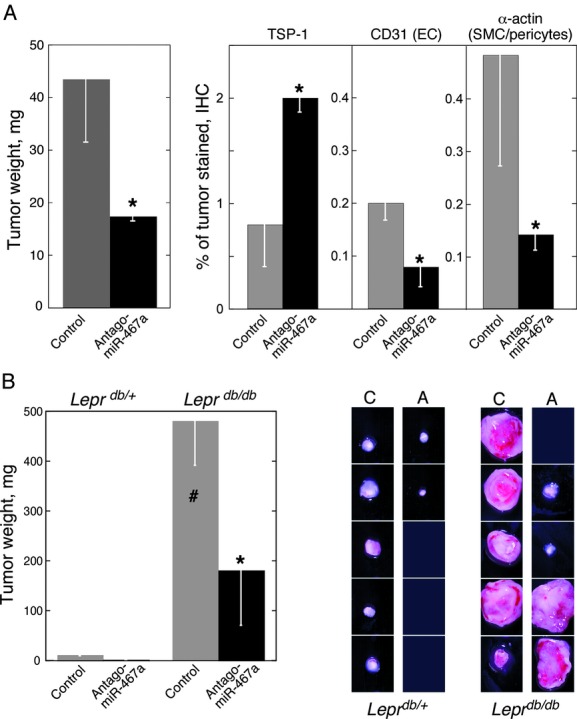
miR‐467 regulates breast cancer growth in *Lepr*^*db/db*^ hyperglycemic mice. A, EMT6 cells stably expressing the antisense antagonist of miR‐467 or a control ribooligonucleotide were injected subcutaneously into STZ‐treated hyperglycemic or normoglycemic mice. Tumors were weighed, stained with anti‐TSP‐1, anti‐CD31, and anti‐α‐actin antibodies. Mean±SEM, *P<0.05 compared with tumors expressing control oligonucleotide (control). Sample size: mice with tumors expressing control ribooligonucleotide (control), n=15; mice with tumors expressing antagomiR‐467, n=16. To quantify immunohistological staining, FOV images were generated and analyzed as described in Methods. B, EMT6 cells stably expressing the antisense antagonist of miR‐467 or a control ribooligonucleotide were injected subcutaneously into hyperglycemic *Lepr*^*db/db*^ or normoglycemic *Lepr*^*db/+*^ mice as described in Methods, and tumors were weighed (in milligrams). Mean±SEM, *P<0.05 compared with tumors expressing control oligonucleotide (control), #*P*<0.05 compared with normoglycemic (*Lepr*^*db/+*^) mice, n=5. Right panel, photographs of individual tumors excised from *Lepr*^*db/db*^ and *Lepr*^*db/+*^ mice: C=EMT6 producing control oligonucleotide; A=EMT6 producing miR‐467 antagonist. STZ indicates streptozotocin; TSP, thrombospondin; FOV, field of view; SEM, standard error of mean.

When EMT6 cells stably transfected with vectors expressing either the antisense antagonist of miR‐467 or a control oligonucleotide were injected into STZ‐treated and normoglycemic mice, the hyperglycemic mice with tumors producing antagomiR‐467 were protected from stimulation of tumor growth by hyperglycemia (Figure 8A). AntagomiR‐467 did not have any effect on TSP‐1 mRNA expression: in both normoglycemic and hyperglycemic tumors expressing the antagonist, TSP‐1 mRNA expression did not differ from that in tumors expressing the control oligonucleotide (Figure S9A). Cell proliferation was assessed in cultured EMT6 cells expressing the antagonist or the control oligonucleotide and incubated in either 5 or 30 mmol/L glucose at 6, 24, and 48 hours. The antagonist did not affect cancer cell growth in vitro (Figure S9B).

Tumors were stained with anti‐TSP‐1, anti‐CD31, and anti‐α‐actin to evaluate the effect of the antagonist on TSP‐1 production and proangiogenic activity. In hyperglycemic mice, the levels of TSP‐1 were increased, and the levels of CD31 and α‐actin were decreased as a result of miR‐467 neutralization (Figure 8A).

### Breast Cancer Tumor Growth Is Increased in Hyperglycemic *Lepr*^*db/db*^ Mice

To confirm that the increased tumor growth and its prevention by the antagonist are not limited to the STZ model of hyperglycemia, we employed a second model, *Lepr*^*db/db*^ mice (Figure 8B). EMT6 cells 1.5×10^6^ were injected subcutaneously in either hyperglycemic or normoglycemic *Lepr*^*db/db*^ and *Lepr*^*db/+*^ mice. The tumors were excised and weighed a week later. Similar to the STZ hyperglycemia model, tumor size was dramatically increased in hyperglycemic *Lepr*^*db/db*^ mice (Figure 8B), and antagomiR‐467 was effective in preventing the hyperglycemia‐induced tumor growth (Figure 8B). miR‐467 levels tended to increase in tumors of *Lepr*^*db/db*^ mice (Figure S10A, B9), and there was a trend of correlation between tumor mass and miR‐467 expression in individual animals (Figure S10A, B10). Tumor mass and miR‐467 level positively correlated with blood glucose level in individual mice (Figure S10B).

## Discussion

The information on specific molecular mechanisms controlled by miRNA is fragmental. In this report, we describe a complete pathway including a specific stimulus (high glucose) inducing an miRNA (miR‐467), a molecular mechanism regulated by this miRNA (translational silencing of TSP‐1 as a result of the direct binding to a specific binding site in 3′ UTR), and a physiological effect (induction of angiogenesis in vivo), resulting in the association of 2 pathologies: hyperglycemia and accelerated angiogenesis and tumor growth. This is the first report that describes involvement of miR‐467 in angiogenesis, the regulation of this miRNA by hyperglycemia, and the physiological function of miR‐467.

miR‐467 is not abundant under basal conditions and is difficult to detect when the levels of glucose are normal. We reported earlier^14^ that the signal leading to TSP‐1 silencing is increased osmolarity. Confirming our published results (equal silencing of TSP‐1 by d‐ and l‐glucose), our new data demonstrated identical effects of d‐glucose, l‐glucose, and sorbitol on miR‐467 upregulation. Hyperosmolarity is a common phenomenon in diabetic patients. When the osmolarity of plasma was assessed in groups of younger and older diabetic patients, the diabetic hyperosmolar state was present in 46% of patients younger than age 30 and 44% of patients older than age 60.^21^ Plasma osmolarity was also increased in hyperglycemic mouse models of diabetes, including STZ‐treated and *Lepr*^*db/db*^ mice.^22–24^ The damaging effects of this clinically relevant condition have not been well addressed, with a very few exceptions (eg, see referene ^25^).

miR‐467 distribution between RNA fractions matched TSP‐1 mRNA distribution: it was translocated to the nonpolysomal fraction in microvascular ECs in response to high glucose. Although circumstantial, this observation further suggested that miR‐467 may participate in translational silencing. Actively translated messages are associated with polysomes, whereas untranslated mRNA is uncoupled from polysomes and found in the nonpolysomal fraction on fractionation. The finding of miR‐467 in the nonpolysomal fraction suggests that it is associated with untranslated messages and may be involved in inhibition of translation and uncoupling of mRNA from polysomes.

To prove that miR‐467 inhibits the production of TSP‐1, we used 2 complementary approaches: transfection of microvascular ECs with miR‐467 to mimic the high‐glucose effect and transfection with antagomiR‐467, the antisense oligonucleotide neutralizing miR‐467,^26–27^ to prevent the silencing effect of high glucose. Results from both approaches confirmed that miR‐467 inhibits TSP‐1 protein production by sequestering TSP‐1 mRNA in the nonpolysomal RNA fraction. The experiments with the constructs, in which the binding site for miR‐467 was mutated, demonstrated that the direct binding of miR‐467 to the 3′ UTR of TSP‐1 is required for the translational silencing of TSP‐1 mRNA in response to high glucose.

The inhibition of TSP‐1 production by miR‐467 was expected to relieve the antiangiogenic pressure of TSP‐1 and to result in increased neovascularization. The regulation of tumor angiogenesis by TSP‐1 is well documented,^28–31^ and miRNA is implicated in this regulation.^28^ However, the previously reported miRNA regulators of TSP‐1 production do not seem to be part of the response to hyperglycemia and hyperglycemia‐induced tumor growth.

First, we used a Matrigel plug as an in vivo angiogenesis model to assess the effect of miR‐467. The Matrigel model allows assessment of angiogenic responses in the absence of uncontrolled influence of the tissues: only vascular and blood cells are invading the plug. In addition, the composition of the matrix can be manipulated, and the transfecting agents can be added (eg, viruses, small RNA). Although this model has limitations (eg, strong inflammatory response, artificial matrix), it has been widely used to examine the vascular responses that have been further translated into more complex models involving multiple tissues (eg, skin wound healing, tumor, ischemia). A cholesterol‐conjugated mimic of miR‐467 was introduced into Matrigel. Cholesterol‐conjugated miRNA and antisense inhibitors have been successfully used both in vitro and in vivo and result in stable and efficient delivery and expression in the target cells.^32–35^ miR‐467 decreased TSP‐1 production and increased the invasion of vascular cells into Matrigel, as was shown by increases in both the number of CD31‐positive and the number of α‐actin‐positive cells. TSP‐1 appears to be the main target of miR‐467 in regulation of angiogenesis: removing TSP‐1 from the pathway by using TSP‐1‐KO mice results in loss of the proangiogenic effect of miR‐467. miR‐467 tended to decrease angiogenesis in TSP‐1‐KO mice, although the difference was not statistically significant. This effect might have resulted from the regulation of other target(s) of miR‐467, which could become more pronounced in the absence of the main target, TSP‐1. As expected, adding exogenous TSP‐1 to the Matrigel plug inhibited angiogenesis and completely prevented the proangiogenic effect of miR‐467.

Thus, using the Matrigel plug model, we documented the effect of miR‐467 on TSP‐1 production and proangiogenic activities in vascular cells and identified TSP‐1 as a main target of miR‐467 in the regulation of angiogenesis. However, TSP‐1 is a secreted protein that is produced by multiple cells in a tissue, and it becomes available to all cell type as a part of the extracellular matrix. Thus, the cumulative effect of hyperglycemia on TSP‐1 levels in a tissue and on angiogenesis would result from the regulation of miR‐467 and TSP‐1 in multiple cell types.

miR‐467 was upregulated in a tissue‐specific manner in hyperglycemic mice. Mouse models of diabetes do not reproduce all features of human disease, and we could not test directly whether miR‐467 upregulation in organs contributes to increased angiogenesis in diabetic animals. However, we were able to test the effect of miR‐467 upregulation in mouse cancer models relevant to the human disease and diabetic complications.

Cancers are associated with diabetes in a tissue‐specific manner. Breast cancer and several other cancers positively correlate with diabetes,^36–40^ whereas prostate cancer has a lower incidence in diabetic patients.^41–47^ Similar to the mechanisms of diabetic tissue‐specific aberrant angiogenesis, the molecular mechanisms of positive or negative tissue‐specific associations of tumor growth and diabetes are unknown. Tumor mass of breast cancer was significantly higher in hyperglycemic animals, but the size of prostate cancer tumors was not increased in diabetic animals. Tumor mass negatively correlated with TSP‐1 level in both cancers, and TSP‐1 transcript level was significantly increased in all cancers in hyperglycemic mice, suggesting that decreased levels of TSP‐1 protein in hyperglycemic breast cancers is a result of posttranscriptional silencing of mRNA. Indeed, the levels of miR‐467 were significantly higher in hyperglycemic breast cancers, suggesting that miR‐467 may regulate TSP‐1 production in breast cancer and microvascular cells, as was suggested by the in vitro study. In contrast, in prostate cancers, higher levels of the transcript resulted in higher levels of the protein. The expression of the miR‐467 antagonist in the breast cancer cells prevented the accelerated growth of tumors caused by hyperglycemia in 2 mouse models of diabetes, confirming that miR‐467 was responsible for the increase in tumor mass and downregulation of TSP‐1 levels in hyperglycemic animals.

Two animal models of hyperglycemia were instrumental in confirming the presence of this novel molecular mechanism in vivo. However, although both diabetic models served well to achieve our simple goal of studying the effects of high glucose in vivo, these mouse models of hyperglycemia and of tumor growth do not fully reproduce the complexity of human diabetes or cancer development in patients. To fully understand the role of this novel molecular mechanism in the pathology of diabetic vascular complications or in the tissue‐specific development of cancer, further studies of human specimens and specific animal models reproducing the features of specific human pathologies will be needed.

This is the first report describing the expression of miR‐467 in humans and a physiological/pathological function for this miRNA. This miRNA controls a novel tissue‐specific pathway of regulation of angiogenesis that may potentially provide a common basis for multiple angiogenesis‐dependent complications of diabetes, including tissue‐specific associations with certain cancers. Our data suggest that miR‐467 plays an important role in the regulation of diabetic angiogenesis and diabetes‐associated tumor growth. miR‐467 is poorly expressed in normoglycemia and can be targeted specifically to effect angiogenesis in a tissue‐specific manner. MicroRNAs and their antisense inhibitors represent a group of small molecules that have tremendous therapeutic potential if the molecular mechanisms are dissected. This breakthrough approach may allow the tissue‐specific correction of aberrant angiogenesis only in organs and tumors in which angiogenesis is increased by hyperglycemia, without affecting physiological angiogenesis or angiogenesis in already ischemic tissues.
